# *OsAPX1* Positively Contributes to Rice Blast Resistance

**DOI:** 10.3389/fpls.2022.843271

**Published:** 2022-03-21

**Authors:** Cong Sheng, Dongli Yu, Xuan Li, Hanxi Yu, Yimai Zhang, Muhammad Saqib Bilal, Hongyu Ma, Xin Zhang, Ayesha Baig, Pingping Nie, Hongwei Zhao

**Affiliations:** ^1^Department of Plant Pathology, College of Plant Protection, Nanjing Agricultural University, Nanjing, China; ^2^Laboratory of Bio-interactions and Crop Health, Nanjing Agricultural University, Nanjing, China; ^3^Institute of Industrial Crops, Shanxi Agricultural University, Taiyuan, China; ^4^Department of Biotechnology, COMSATS University Islamabad Abbottabad Campus, Abbottabad, Pakistan; ^5^College of Life Sciences, Zaozhuang University, Zaozhuang, China

**Keywords:** rice blast resistance, ascorbate peroxidases, *OsAPX1*, miR172a, ROS homeostasis, salicylic acid

## Abstract

Ascorbate peroxidases (APXs) maintain cellular reactive oxygen species (ROS) homeostasis through their peroxidase activity. Here, we report that *OsAPX1* also promotes ROS production such that a delicate cellular ROS homeostasis is achieved temporally after *Magnaporthe oryzae* infection. *OsAPX1* specifically induces ROS production through increasing respiratory burst oxidase homologs (*OsRBOHs*) expression and can be inhibited by DPI, a ROS inhibitor. The time-course experiment data show that the simultaneous induction of *OsAPX1* and *OsRBOHs* leads to ROS accumulation at an early stage; whereas a more durable expression of *OsAPX1* leads to ROS scavenging at a later stage. By the temporal switching between ROS inducer and eliminator, *OsAPX1* triggers an instant ROS burst upon *M. oryzae* infection and then a timely elimination of ROS toxicity. We find that *OsAPX1* is under the control of the miR172a-*OsIDS1* regulatory module. *OsAPX1* also affects salicylic acid (SA) synthesis and signaling, which contribute to blast resistance. In conclusion, we show that *OsAPX1* is a key factor that connects the upstream gene silencing and transcription regulatory routes with the downstream phytohormone and redox pathway, which provides an insight into the sophisticated regulatory network of rice innate immunity.

## Introduction

In plants, reactive oxygen species (ROS) production is considered to be an important defensive response to biotic and abiotic stresses (Jones and Dangl, 2006; Saxena et al., 2016). When plants are subject to biotic stress, elevated ROS production enhances plant resistance to pathogens. For example, after rice stripe virus (RSV) infection, rice *L-ascorbate oxidase* (*AO*) expression was induced, which boosts *planta* ROS accumulation and enhances resistance against RSV (Wu et al., 2017). The accumulation of ROS in rice also plays an important role in resistance against bacterial and fungal pathogens. For example, in rice over-expressing *Triosephosphate Isomerase* (*OsTPI*), ROS accumulated to a significantly higher level than the wild type, dramatically increasing rice resistance against *Xanthomonas oryzae* (Liu Y. et al., 2018). In rice over-expressing *OsWRKY67* or *mitogen-activated protein kinase 15* knockout mutants *Osmpk15*, resistance against *M. oryzae* and *X. oryzae* is enhanced, which is also associated with ROS accumulation (Vo et al., 2017; Hong et al., 2019).

ROS is produced in different plant subcellular compartments (e.g., chloroplasts, mitochondria, peroxisomes, and the apoplastic space) under different stressful conditions (Moller et al., 2007; Wrzaczek et al., 2013; Bose et al., 2014). Pathogen infection-triggered ROS burst happens in apoplast space by the membrane-localized NADPH-dependent oxidase system (also known as respiratory burst oxidase homologs (RBOHs)), which is in contrast to the abiotic stress-triggered ROS that is produced through organelles such as chloroplast, mitochondria, and peroxisome (Apel and Hirt, 2004). In *Arabidopsis*, *rbohd*, and *rbohf* mutants caused susceptibility to bacterial and oomycete infection due to low ROS levels in these mutants (Torres et al., 2002). *OsRBOHb* knockdown plants had lower content of ROS and were more susceptible to rice blast (Nagano et al., 2016).

Although ROS accumulation enhances plant resistance to pathogens, over-production of ROS can lead to detrimental effects such as membrane lipid peroxidation, protein denaturation, carbohydrate oxidation, pigment breakdown, and DNA damage (Moller et al., 2007). To maintain ROS homeostasis, plants have evolved multiple mechanisms to fine-tune ROS homeostasis so that normal biological processes and tolerance/resistance to stresses are delicately balanced. In green plants, ascorbate peroxidases (APXs) are the most significant components of the ROS detoxifying system (Shigeoka et al., 2002; Guan et al., 2015), which play an essential role in controlling intracellular hydrogen peroxide (H_2_O_2_; a ROS species) level (Munné-Bosch et al., 2013; Wang and Chu, 2020). APXs comprise a multigene family that encodes enzymes detoxifying H_2_O_2_. APXs utilize ascorbate as a specific electron donor to convert H_2_O_2_ to water, stabilizing cellular ROS to an ordinary level and maintaining regular metallic processes. For example, *AtAPX1* and *AtAPX2* (cytosolic) protect *Arabidopsis* against diverse abiotic stresses, such as high light, heat, wounding, and drought stress (Panchuk et al., 2005; Mullineaux et al., 2006; Rossel et al., 2006; Hu et al., 2011). In rice, *OsAPX1* was found to be closely linked to chilling and high-temperature stresses (Saruyama and Tanida, 1995). It was reported that high temperature and subsequent chilling could affect *OsAPX1* transcription and activity, indicating a role for *OsAPX1* in the protection of rice seedlings against chilling injury (Sato et al., 2001). In agreement, *OsAPX1*-overexpressing rice also showed enhanced tolerance to chilling at the booting stage (Sato et al., 2011). Besides temperature, the *OsAPX1* transcripts appeared to be up-regulated upon various abiotic stimuli such as wounding, salicylic acid (SA), ethylene, abscisic acid (ABA), H_2_O_2_, copper sulfate, and protein phosphatase (PP) inhibitors but not by jasmonic acid (JA), indicating its involvement in plant tolerance against a broad spectrum of abiotic stresses and stimuli (Agrawal et al., 2003).

Intriguingly, as a ROS scavenger that reduces excessive cellular H_2_O_2_, the induced expression of *APXs* in plants responding to biotic stresses has been widely documented. For example, sunflower *APX1* was dramatically induced by sunflower chlorotic mottle virus (SuCMoV) infection (Rodriguez et al., 2012). In hot pepper, *CaAPX1* was induced upon the Tobacco mosaic virus (TMV) and bacterial pathogen infection (Yoo et al., 2013). In rice, *OsAPX1* and *OsAPX2* transcripts were up-regulated by blast pathogen (*M. oryzae*) attack (Agrawal et al., 2003; Lin et al., 2018). *OsAPX2* was induced in rice by brown planthopper (BPH) (Wei et al., 2009). OsAPX7 and OsAPX1 proteins were significantly accumulated in the resistant but not the susceptible rice line when infected by *Rhizoctonia solani* (Lee et al., 2006; Ma et al., 2019). Another proteomic study found that the OsAPX protein level was constantly induced by *M. oryzae* infection (Narula et al., 2019). Taken together, these observations imply that the induced expression of APXs upon a variety of biotic stresses is not a rare case, although the underlying mechanism is not yet clear.

In this study, we demonstrated that *OsAPX1* plays a double-faced role in both promoting and eliminating ROS accumulation temporally against *M. oryzae* infection. At the early stage of infection, the expression of *OsAPX1* and the membrane-localized ROS producer-*OsRBOHs* are induced, which collectively leads to ROS accumulation. At the later stage of *M. oryzae* infection, *OsRBOHs* expression decline but *OsAPX1* expression remains active, which leads to ROS elimination. This delicate switch turns OsAPX1 from a ROS inducer at the early stage of *M. oryzae* infection into a ROS scavenger at the later stage, which ensures rice deploys a strong ROS burst to confine and eliminate *M. oryzae* right after infection but removes excessive ROS in time before damage to rice cells and tissue occurs. Our results reveal a new facet of *OsAPX1*, which advances our current understanding regarding the function and mechanism of APX. Our discovery is of great significance for understanding the crosstalk between plant responses against biotic and abiotic stresses.

## Materials and Methods

### Pathogen Inoculation

*M. oryzae* Guy11, Js153, and the eGFP-tagged Zhong1 (a gift from Dr. W-M Wang) strains were used for rice infection. *M. oryzae* strains were first inoculated on CM medium (complete medium) that grew at 28^°^C under a 12/12 (light/dark) condition. Spores were collected 2 weeks after inoculation, which were resuspended to 1 × 10^5^ spores ml^–1^. Spores were spray-inoculated on three-leaf-stage rice seedlings. Disease symptoms were examined 5 days later. Genomic DNA was extracted from inoculated leaves for fungal accumulation examination (*MoPot2*; Supplementary Table 1). Disease resistance was also examined by using detached leaves from four-leaf-stage rice. 10 μl spores (1 × 10^5^ spores ml^–1^) were added at two spots of each leaf, and kept in a culture dish with a wet filter. After incubation in a growth chamber at 28^°^C for 24 h in dark, the leaves were kept under a 12/12 h (light/dark) light rhythm for 6 days till disease symptoms were examined. For leaf sheath inoculation, leaf sheaths were prepared from four-leaf-stage rice. 10 μl spores (1 × 10^5^ spores ml^–1^) were inoculated. Fungal hyphae development was observed under a microscope at 24 and 48 h post inoculation (hpi), respectively.

### RNA Blotting

Total RNA extraction was performed as described previously (Zhang et al., 2018). In brief, the inoculated plants were used for RNA extraction at 0, 24, and 48 hpi. Total RNA was extracted using TRIzol reagent (Invitrogen, United States) following the manufacturer’s protocol. RNA was resolved on a 14% denaturing 8 M urea-PAGE gel and then transferred and UV cross-linked onto a Hybond N^+^ membrane (GE Healthcare Life Science, Beijing, China) using UV light. miRNA probes were end-labeled with [γ-^32^P] ATP by T4 polynucleotide kinase (New England Biolabs, Beijing, China). Expression levels were quantified using ImageJ as instructed.

### Generating *OsAPX1* Transgenic Plants

For generating *OsAPX1* overexpression transgenic plants, the full-length CDS was cloned into a pCAM1300 vector driven by a CaMV35S promoter. For the *OsAPX1* silencing mutant, highly specific target regions from the *OsAPX1* CDS were cloned to the pYLCRISPR/Cas9Pubi vector. The construct was transferred to Agrobacterium strain EHA105, which was used for transgenic rice production. For verifying the *OsAPX1* silencing mutant, genomic DNA was used to examine the *OsAPX1* genomic DNA sequence. For verifying *OsAPX1* over-expression plants, total protein samples from the transgenic plant were used for western blot; and total RNA was used to examine the transcript level of *OsAPX1* by qRT-PCR.

### qRT–PCR

1 μg total RNA was reverse transcribed into cDNA by using PrimeScript RT reagent Kit (Takara, Japan). The qRT–PCR was performed in 15 μl of reaction mixture consisting 1.5 μl 10 × SYBR Green (Invitrogen, United States), 1.5 μl PCR buffer, 0.3 μl 10 mM dNTPs (Takara, Japan), 0.3 μl Taq, 0.3 μl ROX DYE2 (Vazyme, China), 1.5 μl 2 mM each primer, and 2 μl appropriate diluted cDNA. The conditions for real-time RT-PCR were as follow: 94^°^C for 3 min, then 40 cycles at 94^°^C for 30 s and 58^°^C for 30 s followed by 72^°^C 35 s for PCR amplification. Transcript levels of each gene were measured by the Applied Biosystems 7500 (Applied Biosystems, United States) according to the manufacturer’s instructions. The data were normalized to the amplification of the rice *18sRNA* gene. Real-time PCR primer sequences are available in Supplementary Table 1.

### Measurement of H_2_O_2_ Accumulation

Leaf tissues and leaf sheaths were dipped into 50 ml solution containing 50 mg Diaminobenzidine (DAB), 25 μl Tween-20 and 2.5 ml 200 mM Na_2_HPO_4_ and vacuum infiltrated for 30 min followed by staining in dark at room temperature (25^°^C) overnight (10 h). The tissues were decolorized in 1:1:1 (v/v/v) acetic acid-ethanol-glycerol solution for 15–20 min at 90–95^°^C and visualized afterward (ThordalChristensen et al., 1997). Decolored leaves or leaf sheath tissues were examined for H_2_O_2_ accumulation around the inoculating loci by using a microscope.

### Measurement of Salicylic Acid Concentrations

The free SA concentration in transgenic rice was measured as described (Liu et al., 2014). The rice tissues were homogenized in liquid nitrogen and then suspended in 90% (v/v) methanol. As an internal standard, 100 mg 3-hydroxy benzoic acid in 100% methanol was added to each sample. The SA solution was filtered and separated on a C18 analytical column using HPLC and detected using fluorescence (excitation at 305 nm, emission at 405 nm; Waters). The HPLC was programmed for isocratic conditions with a flow rate of 0.5 ml/min. The concentration of SA was quantified by area integration of the HPLC peaks.

### Western Blot Analysis

Leaf tissues were snap-frozen in liquid nitrogen and ground into fine powder. The samples were added with 2 × SDS loading buffer, which was boiled at 100°C for 10 min. The supernatant, after 10,000 g centrifugation, was separated by 12% SDS-PAGE gels at 100 V for 1.5 h. The proteins were transferred to PVDF membrane (Bio-RAD, United States), blocked by using 5% dry milk for 30 min, which was followed by Flag antibody (Abmart, China) incubation for 2 h. The membranes were washed by using TBST buffer three times (5 min), followed by 2nd antibody incubation (Abmart, China) for 2 h. The protein signal was detected by chemoluminescence (Tanon, China).

### Enzyme Activity

The APX enzyme activity was examined by using a kit (Beijing Solarbio Science and Technology Co, Beijing, China) measuring the oxidation rate of ascorbic acid within 2 min with a spectrophotometer at 290 nm.

## Results

### *OsAPX1* Expression Is Responsive to *Magnaporthe oryzae* Infection

We challenged 4-week-old rice [the Japonica cultivar Nipponbare (NIP)] with the *M. oryzae* strain Guy11 (compatible strain) and Js153 (incompatible strain) (Supplementary Figure 1A). Compared with the mock, *OsAPX1* expression was significantly induced at 24, 48, and 72 hpi after infection with both strains (Supplementary Figure 1B). At 72 hpi, *OsAPX1* expression increased more than 3 folds. To examine the contribution of *OsAPX1* in rice immunity against the blast disease, we first investigated whether an elevated *OsAPX1* expression would lead to an altered rice blast resistance. Both *OsAPX1* silencing (*cas9-osapx1* #22 and #30; Supplementary Figure 2A) and over-expression (*OsAPX1*-OE #38 and #39; Supplementary Figures 2B,C) transgenic rice were constructed. Genotyping results indicated that *cas9-osapx1* is a homozygous insertion mutant (Supplementary Figure 2A) with a T/C insertion to the 40th nucleotide of the second exon, leading to the complete silencing of *OsAPX1* due to frameshifting. *Cas9-osapx1* lines carried a 1-bp insertion causing protein truncation (Supplementary Figure 2A). Compared to NIP and *cas9-osapx1* #22 rice, the *OsAPX1*-OE #38 rice were significantly higher in growth length, had longer roots (Supplementary Figure 2D), larger seed size (Supplementary Figure 2E), and greater seed weight (per 1,000 seeds) (Supplementary Figure 2F). In contrast, the *cas9-osapx1* #22 rice was shorter and produced smaller seeds both in size and weight. However, neither measurable growth or developmental abnormality nor spontaneous lesions were observable on the leaf surface of the transgenic rice (Supplementary Figure 2D). There was no discernable difference in ROS accumulation between wild type and transgenic rice under normal growth conditions either (Supplementary Figure 2G), indicating that the rice innate immunity is not automatically activated without pathogen infection.

### *OsAPX1* Over-Expressing Plants Are More Resistant to *Magnaporthe oryzae* Infection

**FIGURE 1:**
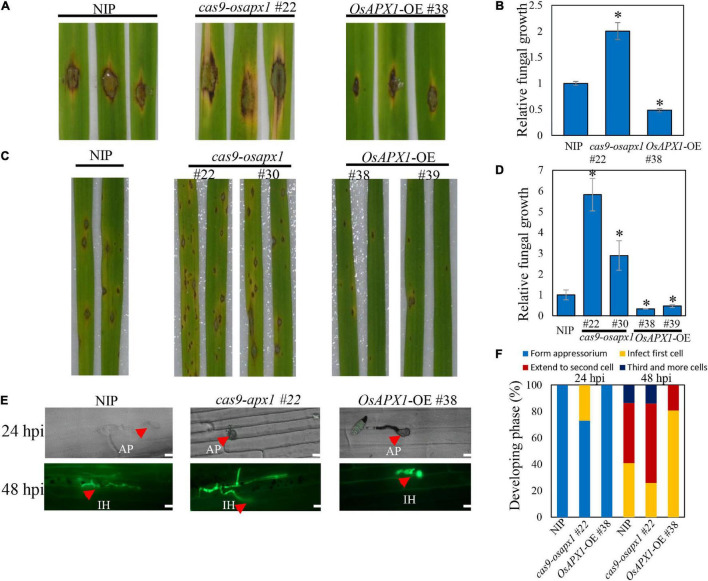
*OsAPX1* overexpression enhances rice resistance to *M. oryzae* Guy11 infection. **(A)** Blast disease assay on the indicated lines. The phenotype of four-leaf stage leaves from wild type and the indicated transgenic lines *cas9-osapx1* and *OsAPX1*-OE plants inoculated by the punch method with spores’ suspension of *M. oryzae* strain Guy11. **(B)** Relative fungal biomass is determined by examining the expression level of *M. oryzae Pot2* gene against *OsUbiquitin* DNA level. Values are means of three replications. Error bars indicate ± *SD.* Asterisks indicate significant differences according to the Student’s *t*-test (*p* < 0.05). **(C)** Rice pathogenicity assays. Leaf phenotypes were observed at 144 h post inoculation (hpi). **(D)** Relative fungal biomass is determined by examining the expression level of *MoPot2* gene against *OsUbiquitin* DNA level at 144 hpi. Values are means of three replications. The error bars indicate ± *SD*. The asterisks indicate significant differences according to the Student’s *t*-test (*p* < 0.05). **(E)** Representative images of sheath cells from the indicated lines infected by eGFP-tagged blast isolate zhong1. Bar = 20 μm. **(F)** Quantification analysis on the progress of fungal infection at 24 and 48 hpi. All of the experiments were repeated three times with similar results.

Detached leaves from transgenic plants were challenged by punching inoculation with Guy11 spores. The *OsAPX1*-OE #38 leaves developed less severe disease symptoms, manifested by significantly smaller necrosis size and lesser discoloration as observed on transgenic leaves when compared to leaves from control plants. Meanwhile, the lesion size on the *cas9-osapx1* #22 leaves was significantly larger than that on the NIP leaves (Figure 1A). The propagation of *M. oryzae* on the infected leaves was quantified by qRT-PCR using primers specific to *MoPot2*, a *M. oryzae* housekeeping gene. In agreement with the resistant phenotype, less hyphae propagation was detected on leaves from the *OsAPX1*-OE #38 than the control, whereas the accumulation of hyphae increased on *cas9-osapx1* #22 (Figure 1B), indicating that over-expression of *OsAPX1* led to enhanced resistance to *M. oryzae* infection.

The association between *OsAPX1* expression level and disease resistance was further confirmed by Guy11 spore-spray inoculation on rice leaves. From 96 hpi on, both NIP and *OsAPX1*-OE or *cas9-osapx1* rice developed typical blast disease symptoms such as scattered lesions on the leaf surface, cell death in the center of some lesions, and chlorosis on some leaves. Specifically, more lesions developed on the *cas9-osapx1* leaves, accompanied by severer chlorosis, than NIP rice. In contrast, both lesion numbers and chlorosis were significantly milder on the *OsAPX1*-OE (#38, #39) leaves, when compared to both *cas9-osapx1* (#22, #30) and NIP rice (Figure 1C). When hyphae growth was quantified, we detected more fungal hyphae on *cas9-osapx1* (#22, #30) rice than both NIP and *OsAPX1*-OE (#38, #39) rice, whereas *OsAPX1*-OE (#38, #39) had the least hyphae development among them (Figure 1D). Our results indicate that *OsAPX1* over-expression led to enhanced resistance against blast fungus infection. When rice sheath epidermal tissue was challenged with an eGFP-labeled *M. oryzae* strain (Zhong-1), we were able to observe and assess infection progress by quantifying the rate of appressorium formation, hyphae development, and invasive hyphae spreading (Figure 1E). At 24 hpi, 27% appressorium developed visible hyphae in *cas9-osapx1* #22 sheath epidermal cells, and 0% hyphae development was recorded on either *OsAPX1*-OE #38 or NIP. At 48 hpi, with hyphae fully developed within the infected cells in all plants, about 60% hyphae were observed to spread to the adjacent *cas9-osapx1* #22 sheath epidermal cells, and about 18% to the 3rd cells; on NIP rice, only about 45% of fungi spread to the adjacent cells and about 13% to the 3rd cells; on *OsAPX1*-OE #38 rice, only about 20% fungi spread to adjacent cells but none of them spread to a 3rd cell (Figure 1F). Our results indicate that *OsAPX1* expression level is positively related to rice ability resisting fungal hyphae development in infected tissue.

### *OsAPX1* Is Induced by *Magnaporthe oryzae* Infection at Transcript Level *via* a miR172a-OsIDS1 Regulatory Module

**FIGURE 2:**
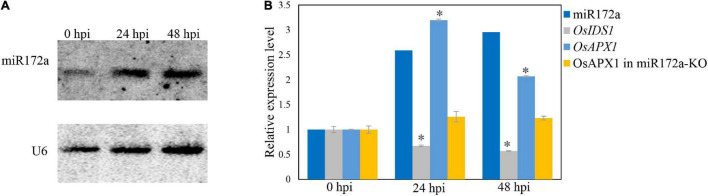
*M. oryzae* induces *OsAPX1* transcription *via* a *miR172a-OsIDS1* regulatory module. **(A)** RNA-blotting detection of mR172a at the indicated time points upon *M. oryzae* infection. U6 was used as a loading control. **(B)** miR172a relative abundance (from **A**) and qRT-PCR analysis of *OsIDS1* and *OsAPX1* expression (in both NIP and miR172a-KO rice) at the indicated time points upon *M. oryzae* infection. qRT-PCR values are means of three replications. Error bars indicate ± SD. Student’s *t*-test was used to determine the significance of differences between 0 hpi and the indicated time points. Asterisks indicate significant differences (*p* < 0.05). The qRT-PCR experiments were repeated three times with similar results.

It was reported that the *OsAPX1* promoter is bound by transcription factor INDETERMINATE SPIKELET1 (OsIDS1), which inhibits the expression of *OsAPX1* (Cheng et al., 2021). *OsIDS1* is a target of miR172a, which silences *OsIDS1* by reverse-complementary sequence match (Cheng et al., 2021). To check whether the induced expression of *OsAPX1* is a result of variated expression of miR172a, we examined miR172a expression by a reverse complementary DNA probe through northern blot. We found that the expression of miR172a was induced significantly at 24 and 48 hpi (Figure 2A). The expression level of *OsIDS1* decreased at both 24 and 48 hpi, while the expression of *OsAPX1* increased at both 24 and 48 hpi, correspondingly (Figure 2B). If miR172a expression was inhibited (miR172a-KO), the expression of *OsAPX1* did not significantly change along with *M. oryzae* infection further (Figure 2B).

**FIGURE 3:**
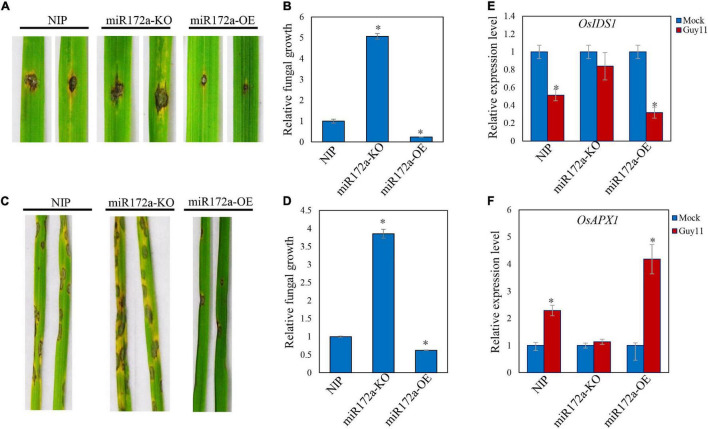
miR172a enhances rice resistance to *M. oryzae*. **(A)** Punch inoculation of *M. oryzae* strain Guy11 on four-leaf-stage leaves from wild type (NIP) and the indicated transgenic lines miR172a-KO and miR172a-OE. **(B)** Relative fungal biomass is determined by examining the expression level of *MoPot2* gene against *OsUbiquitin* DNA level. Values are means of three replications. Error bars indicate ± *SD*. Asterisks indicate significant differences according to the Student’s *t*-test (*p* < 0.05). **(C)** Rice pathogenicity assays. Leaf phenotypes were observed at 144 hpi. **(D)** Relative fungal biomass is determined by examining the expression level of *MoPot2* gene against *OsUbiquitin* DNA level. Values are means of three replications. Error bars indicate ± *SD*. Asterisks indicate significant differences according to the Student’s *t*-test (*p* < 0.05). All of the experiments were repeated three times with similar results. **(E)** *OsIDS1* gene expression level in NIP, miR172a-KO, and miR172a-OE plants after *M. oryzae* infection. Values are means of three replications. Error bars indicate ± *SD*. Asterisks indicate significant differences according to the Student’s *t*-test (*p* < 0.05). **(F)** *OsAPX1* gene expression level in NIP, miR172a-KO, and miR172a-OE plants after *M. oryzae* infection. Values are means of three replications. Error bars indicate ± *SD*. Asterisks indicate significant differences according to the Student’s *t*-test (*p* < 0.05).

We inoculated Guy11 spores on the detached leaves of both wild type and miR172a transgenic rice (a gift from Cheng) (Cheng et al., 2021). The lesions on the miR172a-OE rice leaves were significantly smaller than the WT rice. In contrast, miR172a-KO leaves developed significantly larger lesions than WT rice. Cell death and chlorosis were also very obvious on miR172a-KO leaves (Figure 3A). Guy11 developed more fungal mass on the miR172a-KO rice, while much lesser on the miR172a-OE leaves than the WT rice (Figure 3B). When the rice was spray-inoculated with Guy11 spores, there was almost no lesions development on miR172a-OE rice leaves, whereas miR172a-KO rice exhibited much more lesions than both the WT and miR172a-OE rice (Figure 3C). Quantification of hyphae in leaves also indicated that Guy11 was spread much more in tissues of miR172a-KO rice than the WT and miR172a-OE rice (Figure 3D). Compared with mock, the *OsIDS1* and *OsAPX1* gene expression levels were not significantly changed after *M. oryzae* infection in the miR172a-KO plant, but in the miR172a-OE plant, the expression of *OsIDS1* reduced significantly and *OsAPX1* was induced significantly (Figures 3E,F). In summary, our results indicate that *OsAPX1* is subject to a miR172a-*OsIDS1* regulatory module upon *M. oryzae* infection.

### *OsAPX1* Temporally Fine-Tunes Reactive Oxygen Species

**FIGURE 4:**
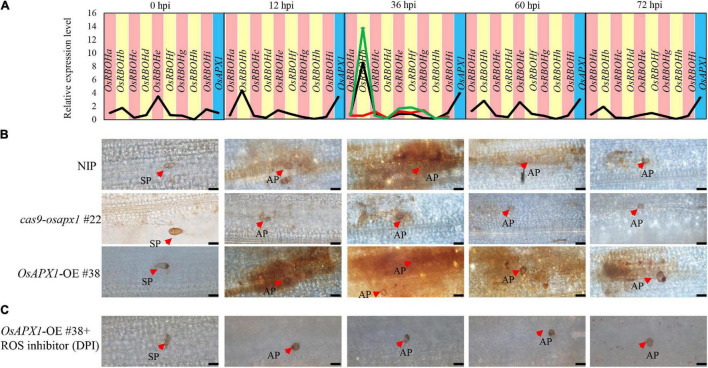
*OsAPX1* temporally fine-tunes ROS. **(A)** *OsRBOH* genes expression level at the indicated time points by *M. oryzae* infected in NIP. Red-line in 36 hpi indicates *OsRBOHs* expression level in *cas9-osapx1* rice at the time points infected by *M. oryzae*. Green-line in 36 hpi indicates *OsRBOHs* expression level in *OsAPX1-*OE rice at the time pointed infected by *M. oryzae.* Values are means of three replications. Error bars indicate ± SD. **(B)** ROS accumulation at the indicated time pointed by *M. oryzae* infected in the transgenic plant. Red arrows indicate infection loci. Bar = 20 μm. **(C)** The pictures show ROS accumulation at the infection sites of the *OsAPX1*-OE rice leaves at the indicated time pointed by DPI treatment. Red arrows indicate infection loci. Bar = 20 μm. All of the experiments were repeated three times with similar results.

ROS production is a hallmark of plant early defense responses, which represents a successful pathogen recognition and the activation of plant defense response (Torres, 2010; Nie et al., 2017; Qi et al., 2017; Zhang et al., 2018). We checked the ROS accumulation in both transgenic and control plants. We found that ROS accumulation in *OsAPX1*-OE #38 and NIP plants could be observed around infection sites at 12 and 36 hpi, then reduced at 60 and 72 hpi. *OsAPX1*-OE #38 rice obviously accumulated more ROS at the penetrating sites than the NIP rice, whereas it was the least observed in *cas9-osapx1* #22 rice (Figure 4B). The observation that Guy11 infection led to more ROS accumulation in *OsAPX1*-OE #38 rice than in WT rice challenges our current knowledge that *OsAPX1* eliminates, instead of, induces ROS accumulation. However, numerous groups have reported that elevated *APX* expression is associated with ROS accumulation (details in discussion). We, therefore, hypothesized that there must be a mechanism that ROS accumulation and scavenging coalesce.

Diphenyleneiodonium (DPI) is a flavoenzyme inhibitor that prevents the activation of NADPH oxidases necessary for ROS generation in plants (Cross and Jones, 1986; Li et al., 2020a). We found that ROS accumulation around the infection loci was dramatically reduced in *OsAPX1*-OE #38 rice after DPI treatment (Figure 4C), suggesting that these ROS may be produced by *OsRBOHs*.

*OsRBOHs* are crucial components in ROS accumulation upon rice *M. oryzae* infection (Dangol et al., 2019). OsRBOHs reduce cellular oxidation potential by catalyzing the transfer of electrons from NADPH to oxygen (O_2_), which generates superoxide radicals (O^∙2–^). A previous study reported that the lack of *sAPX* and *tAPX* drastically decreased the expression of H_2_O_2_ responsive genes in *Arabidopsis* under photooxidative stress (Maruta et al., 2010). Under flood stress, *AtSUS1*, *AtPEPC*, *AtLDH* gene expression increased in *Arabidopsis* plant that overexpression sponge gourd *APX* (*LcAPX*) (Chiang et al., 2017). Therefore, we hypothesized that *OsAPX1* affects *OsRBOHs* gene expression under *M. oryzae* infection. To verify our hypothesis, we checked the *OsRBOHs* expression level in transgenic plants at 36 hpi, the time point when ROS accumulation peaked. Compared to NIP, *OsRBOHs* (especially *OsRBOHb*) expression was significantly induced in *OsAPX1*-OE #38 rice (green line) and significantly reduced in *cas9-osapx1* #22 rice (red line) (Figure 4A). These results suggest that *OsAPX1* may influence ROS production by affecting *OsRBOH* genes expression when rice is infected by *M. oryzae*.

The generation and removal of ROS are two parallel activities that maintain cellular ROS homeostasis (Li et al., 2016). We found that the expression of *OsAPX1* (ROS removal) was constantly induced after *M. oryzae* infection, but ROS accumulation was temporal. Therefore, we speculated that ROS generation gene *OsRBOHs* expression is temporally induced. Indeed, the time-course experiment showed that *OsRBOHs* gene expression level was temporal during *M. oryzae* infection. At 0 hpi, *OsRBOHb*, *OsRBOHe*, and *OsRBOHi* expression were detectable, among which *OsRBOHe* is the major contributor of expressed *OsRBOHs*. Upon *M. oryzae* infection, *OsRBOHb* became a major contributor, which was induced at 12 hpi and peaked at 36 hpi. From 60 hpi on, expression of *OsRBOH* genes declined, especially *OsRBOHb*. It should be noticed that at the same infection period the expression of *OsAPX1* was also dramatically induced (Figure 4A). The result indicates that *OsAPX1* and *OsRBOHb* were simultaneously induced at the early stage after *M. oryzae* infection; at the later stage, the expression of *OsAPX1* was constantly induced; however, *OsRBOHb* was declined. We also checked APX enzyme activity in NIP and transgenic plants. We found that APX enzyme activity was elevated both in the early and late-stage after *M. oryzae* infection (Supplementary Figure 3). In summary, these results showed that ROS generation activity masked ROS scavenging activity at the early stage after *M. oryzae* infection, which led to ROS accumulation; however, ROS scavenging activity prevailed at the later stage, which eventually led to ROS removal.

### Salicylic Acid but Not Jasmonic Acid Signaling Pathway Activates in *OsAPX1*-OE Rice

**FIGURE 5:**
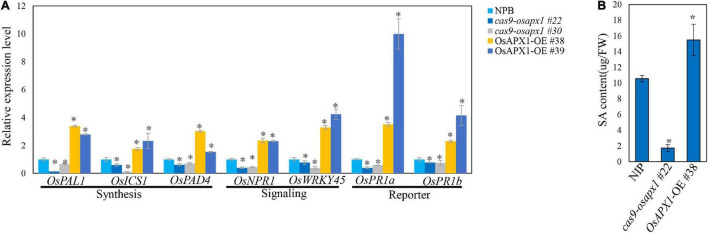
Overexpression of *OsAPX1* enhanced SA signaling pathway gene expression and SA content. **(A)** The expression level of SA signaling pathway relative genes are compared between *OsAPX1* transgenic rice and wild type rice by qRT-PCR. Values are means of three replications. Error bars indicate ± *SD*. Asterisks indicate significant differences according to the Student’s *t*-test (*p* < 0.05). **(B)** The content of free SA in *OsAPX1*-OE, *cas9-osapx1*, and WT rice is measured by using HPLC. The *OsAPX1*-OE rice appears to accumulate a higher level of free SA than the WT and *cas9-osapx1* rice. Measurement is repeated three times, error bars indicate ± SD, and asterisks indicate significant differences between samples according to the Student’s *t*-test (*p* < 0.05). All of the experiments were repeated three times with similar results.

ROS are crucial signal molecules that can activate phytohormone signaling pathways such as the salicylic acid and jasmonic acid pathway (Torres, 2010; Xu et al., 2015), which play important roles in plant innate immunity (Liu et al., 2016; Hong et al., 2019). To further investigate the mechanism underlying *OsAPX1*-mediated disease resistance against rice blast, we examined the expression of several key SA and JA signaling pathway genes. *OsPR1a* and *OsPR1b* are important SA signaling pathway reporter genes, which are induced by many pathogen infections. Our results revealed that in *OsAPX1*-OE rice the expression of *OsPR1a* was around 3-fold and 9-fold higher than that in NIP, whereas *OsPR1b* expression was around 2-fold and 4-fold higher than that in NIP (Figure 5A), indicating that SA signaling pathway was activated when *OsAPX1* is over-expressed. Genes involved in SA synthesis and signal transduction were also checked. As shown in Figure 5A, genes associated or directly participated in SA synthesis such as *OsPAD4*, *OsICS1*, and *OsPAL1* were significantly induced in the *OsAPX1*-OE rice, among which *OsPAD4* showed an around the 3- and 1.5-fold increase. Expression of genes involved in SA signaling transduction, such as *OsNPR1* and *OsWRKY45*, was also increased in *OsAPX1*-OE rice (Figure 5A). The expressions of these genes were reduced in the *cas9-osapx1* rice (Figure 5A). Taken together, our results indicate that components involved in SA synthesis, signaling transduction are induced upon *OsAPX1* over-expression, which may contribute to enhanced disease resistance.

To confirm the relationship between *OsAPX1* and the enhanced expression of SA signaling pathway components, we further examined *in vivo* SA concentration in WT and transgenic plants by HPLC analysis. Free SA concentration was about 10 μg in each gram of fresh tissue (μg/FW) in NIP and was more than 15 μg/FW in *OsAPX1*-OE rice, which is about 1.5 times as much as in the NIP rice. In contrast, SA content in *cas9-osapx1* rice was about 1.72 μg/FW, which is much lower than in the NIP rice (Figure 5B).

We also examined the expression of *OsPDF1.2*, the reporter gene of the JA signaling pathway. The expression of *OsPDF1.2* was not changed measurably in *OsAPX1*-OE or *cas9-osapx1* rice (Supplementary Figure 4), indicating that the JA signaling pathway was not affected by *OsAPX1*. In agreement, the expression of most key components participating in JA biosynthesis (e.g., *OsLOX5*, and *OsAOS2*) and signal transduction (e.g., *OsJAZ8*, *OsCOL1b*, *OsMYC2*) were not significantly changed between control and both *OsAPX1*-OE and *cas9-osapx1* rice (Supplementary Figure 4), indicating that JA signaling pathway is not a major contributor to the *OsAPX1*-mediated defense against *M. oryzae* infection.

### OsAPX1 May Play a Role in Broad-Spectrum Resistance

**FIGURE 6:**
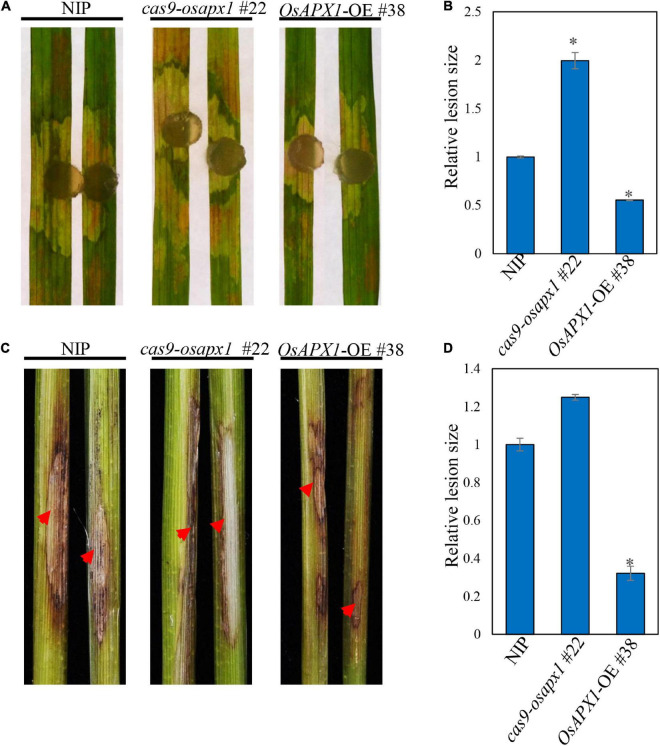
*OsAPX1* positively regulates resistance to *Rhizoctonia solani.* **(A)** The disease phenotypes of the leaves of *R. solani* infected NIP, *cas9-osapx1*, and *OsAPX1*-OE at 48 hpi using a mycelium plug. **(B)** The relative lesion sizes were measured using ImageJ. Values are means of two replications. Error bars indicate ± SD. Asterisks indicate significant differences between samples according to the Student’s *t*-test (*p* < 0.05). **(C)** The phenotypes of the stem of *R. solani* infected NIP, *cas9-osapx1*, and *OsAPX1*-OE at 15 days post inoculation (dpi). **(D)** The relative lesion sizes were measured using ImageJ. Values are means of two replications. Error bars indicate ± SD. Asterisks indicate significant differences between samples according to the Student’s *t*-test (*p* < 0.05). Red arrows indicate infection loci. All of the experiments were repeated three times with similar results.

We also tested whether *OsAPX1* responds to another rice disease. *R. solani* causes rice sheath blight, which is one of the devastating rice diseases. We challenged both transgenic and WT rice with *R. solani*. *Cas9-osapx1* #22 rice exhibited significantly larger lesions and server chlorosis than the NIP rice, whereas *OsAPX1*-OE #38 rice had smaller lesions and weaker chlorosis (Figure 6A), indicating OsAPX1 may also play a role in resistance to sheath blight. Lesion size quantification clearly demonstrated a positive relationship between *OsAPX1* expression level and disease symptoms (Figure 6B). When the rice was challenged by stem-inoculated *R. solani*, *OsAPX1*-OE #38 rice exhibited significantly smaller lesions than that from the NIP rice at 15 dpi, whereas *cas9-osapx1* #22 leaf sheath had significantly larger lesions than the NIP rice (Figure 6C). Lesion size quantification confirmed our judgment that the expression level of *OsAPX1* is positively related to resistance to sheath blight disease (Figure 6D).

## Discussion

It has been recognized that APXs can reduce excessive intracellular ROS elicited by different sorts of abiotic stresses, leading to an enhanced tolerance in different plant species (Ribeiro et al., 2012; Zhang et al., 2013; Guan et al., 2015; Yan et al., 2016). According to our previous knowledge, APXs’ function is associated with their peroxidase activity. In this study, we demonstrate that OsAPX1, together with OsRBOHb, maintains cellular ROS status by temporally balancing ROS generation and elimination, thereby enhancing rice resistance to rice blast. Our study reveals a delicate manipulation of cellular ROS homeostasis, which ensures rice battles off fungal pathogens at an early stage while protecting itself from excessive oxidative stress. Our discovery may lead to an in-depth understanding of APXs responding to both biotic and abiotic stresses.

### *OsAPX1* Regulates Reactive Oxygen Species Production by Affecting *OsRBOHs* Expression

Reactive oxygen species (ROS) burst is an important defense response upon pathogen infection, in which APX is supposed to play a ROS scavenger role. It appeared intriguing to us at the very beginning that *OsAPX1*-OE rice accumulates significantly more H_2_O_2_ than the WT plants while *cas9-osapx1* showed the least H_2_O_2_ (Figure 4B). We later found that other groups also reported similar observations. For example, it was reported that SuCMoV-infected sunflower leaves demonstrated simultaneously increased expression of *APX1* and elevated H_2_O_2_ accumulation (Rodriguez et al., 2012). In an *NPR1*-silencing tomato line that is highly resistant to *Botrytis cinerea*, both APX activity and H_2_O_2_ accumulation increased (Li et al., 2020b). Applying polyamine to apricot fruits not only enhanced resistance to black spot disease but also induced transcriptional expression of *PaAPX* and H_2_O_2_ accumulation (Li et al., 2019b). *R. solani*-infected beans exhibited both boosted APX activity and H_2_O_2_ accumulation during its infection (Keshavarz-Tohid et al., 2016). All these reports recorded simultaneous increases in both APX expression and ROS accumulation in multiple species.

When we carefully investigated the origin of ROS, we found that most of the ROS accumulation was demolished in the *OsAPX1*-OE plant by DPI treatment (Figure 4C). DPI is a flavoenzyme inhibitor that specifically prevents the activation of NADPH oxidases required for ROS generation (Cross and Jones, 1986). RBOHs are membrane-localized NADPH-dependent oxidases that catalyze the production of superoxide from oxygen and NADPH (Kaur et al., 2014; Li et al., 2019a). Increased *OsRBOHs* expression level very likely led to elevated ROS production. Taken together, we are confident to conclude that *OsAPX1* contributes to cellular ROS homeostasis after *M. oryzae* infection. Our results may also explain the observation made on other APXs. For example, *N. benthamiana* leaves over-expressing sugarcane APX (*ScAPX6*) accumulated significantly more H_2_O_2_ and was more resistant to *Fusarium solani var. coeruleum* infection (Liu F. et al., 2018).

Our study observed that *OsRBOHs* genes expression levels increased in the *OsAPX1*-OE plant. The phenomenon is similar to other studies. For example, overexpressing *ScAPX6* in *N. benthamiana* leaves, result in *NtPR1a, NtPR3*, and *NtEFE26* gene expression levels were increased after *Fusarium solani* var. *coeruleum* infection (Liu F. et al., 2018). Transcriptome analysis showed that lignin biosynthesis relative genes induced in *Rheum austral APX* overexpression line under salt stress (Shafi et al., 2015). However, the mechanism of gene expression level increase needs further study.

### *OsAPX1* Temporally Regulates Reactive Oxygen Species Production

We found both *OsAPX1* and *OsRBOHs* were involved in ROS homeostasis management. Both expressions were induced almost simultaneously, but the induced expression of *OsRBOHs* terminated shortly after *M. oryzae* infection while the expression of *OsAPX1* was sustained. Previous studies reported that plant APX activity increases and more ROS accumulation were found under biotic stress (Rodriguez et al., 2012; Liu F. et al., 2018). It indicates that ROS generation and elimination co-exist at the same time, and ROS accumulation was temporal during the *M. oryzae* infection (Figure 4B). From 0 to 36 hpi, both *OsRBOHs* (especially *OsRBOHb*) and *OsAPX1* are induced, during this period, the peroxidase activity is masked by the oxidase activity such that ROS homeostasis leans toward production rather than degradation. After 36 hpi, the expression of *OsRBOHb* gradually begins to decline but *OsAPX1*’s peroxidase activity remains strong, which favors ROS degradation over its production. The overlapping expression between *OsRBOHs* (especially *OsRBOHb*) and *OsAPX1* corresponds very well with cellular ROS accumulation patterns (Figure 4A), manifesting a significant role played by *OsRBOHb*. This is further supported by the failed ROS accumulation in *cas9-osapx1* rice. Although some OsRBOH members expressed normally, or even slightly increased, reduced *OsRBOHb* expression played a dominant role and led to failed overall ROS accumulation.

Therefore, we concluded that it is the unique expression patterns of *OsRBOH* and *OsAPX1* that is governing the ROS rhythmic generation and elimination upon *M. oryzae* infection. At the early stage of *M. oryzae* infection, *OsAPX1* and *OsRBOHb* co-expressed. OsAPX1’s peroxidase activity was masked by OsRBOH activity, which leads to ROS accumulation; at the later stage, *OsRBOHb* expression declined while *OsAPX1* expression remained constantly activated, which led to ROS elimination. This hypothesis was further supported by the DPI treatment that specifically inhibits ROS. DPI treatment functionally mimics the earlier termination of RBOH activity. In *OsAPX1*-OE rice, DPI treatment destroys RBOH activity such that OsAPX1 scavenger activity was unmasked, demonstrated by the absence of ROS accumulation around the infection loci (Figure 4C). In contrast, when the OsRBOH activity was not offset by the DPI treatment, ROS accumulation peaked at 36 hpi before it dropped.

Our study showed that *OsAPX1* can induce *OsRBOHs* expression after *M. oryzae* infection. The dynamic *OsRBOHb* expression was also reported, in which *OsRBOHb* was induced at 24 hpi but then declined at 48 hpi upon *M. oryzae* infection (Yang et al., 2017). It was reported that *OsRBOHs* expression could be regulated by other factors. For example, OsEIL1 binds *OsRBOHb* promoter and regulates its expression (Yang et al., 2017). *OsHXK1* can regulate *OsRBOHs* gene expression through an unknown mechanism (Zheng et al., 2019); auxin can induce *OsRBOHs* expression (Zhang et al., 2019). Therefore, we speculate that the *OsRBOHs* expression pattern in responding to *M. oryzae* infection is intricately regulated, in which *OsAPX1* plays an unstated role.

### *OsAPX1* Expression Is Sophisticatedly Regulated

We previously showed that OsAPX1 proteins were induced by *M. oryzae* infection as early as 24 hpi (Lin et al., 2018). In this study, we further confirmed that *OsAPX1* was induced at the early infecting stage at transcription level as well (Figure 2B and Supplementary Figure 1B), suggesting that *OsAPX1* participates in blast resistance from a very early stage. The induced expression of *OsAPX1* and other rice APXs has been reported by multiple groups. For example, Agrawal and colleagues revealed that *OsAPX1*/*2* transcripts were up-regulated by *M. oryzae* infection (Agrawal et al., 2003). *OsAPX1*/*2* were differentially expressed by some defense-related phytohormone treatments (Durner and Klessig, 1995; Chandrashekar and Umesha, 2014). OsAPX1 protein is induced by *R. solani* in resistant cultivars but not in susceptible cultivars (Ma et al., 2019). OsAPX7 protein was significantly induced by a necrotrophic pathogen, *R. solani* (Lee et al., 2006). Similarly, *OsAPX8* transcription was dramatically induced when rice was infected by *Xanthomonas* (Jiang et al., 2016). However, the mechanism underlying these inductions is not clear to date.

Our study showed that *OsAPX1* expression upon *M. oryzae* infection is regulated by the *miR172a*/*OsIDS1* module. miR172a has been reported to play a role in immunity, such as in tomato (*Solanum lycopersicum*), in which over-expressing miR172a and miR172b enhance resistance to *Phytophthora infestans* by inhibiting the expression of AP2/ERF (Luan et al., 2018). Immunity-related genes expressed significantly higher in miR172b-OE *Arabidopsis* than in wild type after flg22 treatment (Zou et al., 2020). The above evidence supports the notion that miR172 is a positive immune regulator in multiple plants. In this study, we found that miR172a was significantly induced after *M. oryzae* infection (Figure 2A), accompanied by reduced expression of *OsIDS1*, its target gene. Most interestingly, the fluctuated expression of miR172a and *OsIDS1* correspond very well with *OsAPX1*, their downstream target gene (Figure 2B). Our results are consistent with an observation made by Cheng and her colleagues that miR172 was induced by salt stress and contributes to salt stress through *miR172a*/*OsIDS1* module (Cheng et al., 2021). Whether rice resistance/tolerance converges at miR172a/IDS1/*OsAPX1* module is worth further exploration.

### *OsAPX1* Affects Defense Responses Through Salicylic Acid Signaling Pathway

Other than the direct elimination of pathogens, ROS also serves as a signal molecule that is involved in multiple innate immunity signaling pathways, including SA and JA. SA and JA signaling pathways are an important way to respond to biotic stress (Van Camp et al., 1998; Quan et al., 2008; Torres, 2010; Xu et al., 2015; Tian et al., 2016). In the current study, we were able to show that the JA signaling pathway was not changed measurably (Supplementary Figure 4). Instead, several key components involved in both SA synthesis and signaling were upregulated in *OsAPX1*-OE plants but were downregulated in *cas9-osapx1* plants. *OsPAD4*, *OsPAL1*, and *OsICS1* are genes involved in SA synthesis, among which *OsPAD4* is involved in SA regulation whereas *OsPAL1* and *OsICS1* are the two key components directly involved in SA synthesis. It was proposed that *OsICS1* is an important factor that contributes to most of the induced SA production upon biotic challenge (Wildermuth et al., 2001; Loake and Grant, 2007). Consistently, in *OsAPX1*-OE plants we observed a remarkable stronger induction of *OsICS1* than *OsPAL1*, indicating *OsAPX1* contributes to the risen SA synthesis primarily through *OsICS1* induction (Figure 5A). Key components involved in SA signaling such as *OsNPR1* and *OsWRKY45* were also differentially expressed in *OsAPX1*-OE plants. In *OsAPX1*-OE plants, both *OsNPR1* and *OsWRKY45* branches were up-regulated. In *cas9-osapx1* plants, SA signaling pathway components behaved to an opposite profile as in the *OsAPX1*-OE plants, indicating a reliable connection between *OsAPX1* and SA signaling pathway. The induction of *OsWRKY45* was slightly stronger than the expression of *OsNPR1* (Figure 5A), suggesting that *OsWRKY45* is favored by *OsAPX1*.

We observed that *OsAPX1*-OE leaves had much higher SA content, whereas *cas9-osapx1* leaves had much lower SA contents, compared to the WT plants (Figure 5B). Rice accumulates high basal levels of SA (8–37 μg g^–1^ fresh weight) that do not change significantly upon pathogen attack (Silverman et al., 1995), which leads to the misconception that the SA signaling pathway is unrelated to rice defense against blast infection. High basal endogenous SA content does not mean that rice is insensitive to SA signaling. For example, exogenously administrated SA triggers resistance to *M. oryzae* in adult plants (Iwai et al., 2007). In rice mutant with constantly activated SA signaling pathway (i.e., *osmpk15*), rice resistance against blast disease is enhanced (Hong et al., 2019). Moreover, synthetic SA analogs such as probenazole, benzothiadiazole (BTH), and tiadinil can induce rice defense response to a wide range of pathogens, ranging from (hemi) biotrophic *M. oryzae* and bacterial leaf blight pathogen *X. oryzae pv. oryzae* (*Xoo*), to necrotrophic root pathogens such as *Hirschmanniella oryzae* (Shimono et al., 2007; De Vleesschauwer et al., 2008, 2012; Nahar et al., 2012; Xu et al., 2013).

**FIGURE 7:**
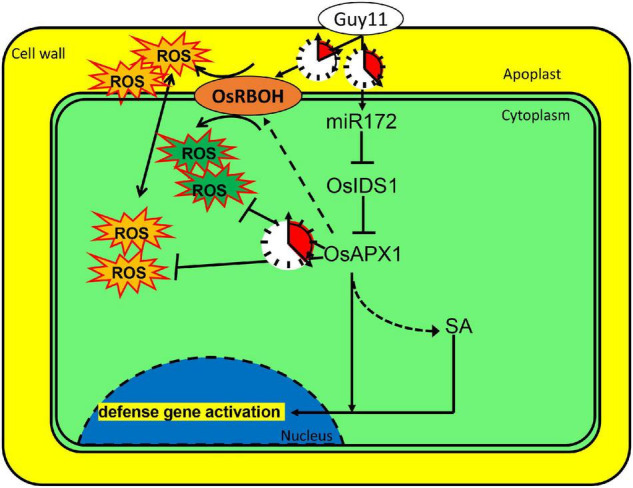
*OsAPX1* manipulates cellular ROS homeostasis upon *M. oryzae* infection. A model demonstrating how OsAPX1 might be involved in rice resistance against the blast disease. Guy11 infection induces miR172a expression, which releases *OsAPX1* expression from the restriction of a transcription factor, OsIDS1. *OsAPX1* overexpression increase *OsRBOHs* expression in the early stage that promotes ROS production. ROS is eliminated by OsAPX1 at a later stage that protects rice from its toxicity. OsAPX1 also activates both SA synthesis and signaling, which leads to downstream defense gene activation.

Based on the results, we propose that rice can sense *M. oryzae* infection and induce the expression of a set of immune regulating factors. miR172a was induced upon *M. oryzae* infection. The induced expression of miR172a leads to the suppressed expression of *OsIDS1*, which encodes a transcription factor and enhances *OsAPX1* transcription as well. Meanwhile, *OsRBOHb* was induced at the early stage, but it was reduced at the later stage. At the early stage, OsRBOH activity masked OsAPX activity that results in ROS generation; at the later stage, OsAPX activity was unmasked by OsRBOH activity that leads to ROS elimination. By a delicately regulated sequential expression, *OsAPX1* and *OsRBOHs* manipulate ROS homeostasis temporally. ROS accumulates shortly after *M. oryzae* infection, which is at the earliest time and the most imminent frontier. After the initial ROS burst, *OsAPX1* removes excessive ROS to prevent the rice from ROS toxicity (Figure 7). It would be interesting to investigate whether *OsAPX1* plays a role in tolerance against abiotic stresses employing a similar mechanism.

## Data Availability Statement

The original contributions presented in the study are included in the article/Supplementary Material, further inquiries can be directed to the corresponding author/s.

## Author Contributions

CS and HZ designed the experiments and wrote the manuscript. CS, DY, XL, HY, YZ, XZ, MS, AB, HM, and PN performed the experimental work. CS, AB, and HZ performed the data analysis.

## Conflict of Interest

The authors declare that the research was conducted in the absence of any commercial or financial relationships that could be construed as a potential conflict of interest.

## Publisher’s Note

All claims expressed in this article are solely those of the authors and do not necessarily represent those of their affiliated organizations, or those of the publisher, the editors and the reviewers. Any product that may be evaluated in this article, or claim that may be made by its manufacturer, is not guaranteed or endorsed by the publisher.

## Supplementary Material

Supplementary Figure 1 *OsAPX1* transcript induces by *M. oryzae* infection. **(A)** Rice (*Oryzae sativa* cv. Nipponbare) infected with strain Guy11 and Js153. The disease symptom is recorded at 72 hpi. **(B)** Relative transcriptional level of *OsAPX1* in rice infected by mock, Guy11, and Js153 at 24, 48, and 72 hpi (*OsAPX1/18srRNA*). Values are means of three replications. Error bars indicate ± SD. Asterisks indicate significant differences between samples according to the Student’s *t*-test (*p* < 0.05). All of the experiments were repeated three times with similar results.

Supplementary Figure 2 Validation of *OsAPX1* transgenic rice. **(A)** Sequence confirmation of the homogenous *cas9-osapx1* mutant lines. **(B)** Protein confirmation of *OsAPX1*-OE lines. **(C)** *OsAPX1* transcript level in *OsAPX1*-OE and NIP rice. Values are means of three replications. Error bars indicate ± SD. Asterisks indicate significant differences between samples according to the Student’s *t*-test (*p* < 0.05). **(D)** The phenotype of the NIP, *cas9-osapx1*, and *OsAPX1*-OE rice at 45 days post-sowing. **(E)** Comparison of grain length in *OsAPX1*-OE, *cas9-osapx1*, and NIP rice. **(F)** Weight of 1,000 seeds of *OsAPX1*-OE, *cas9-osapx1*, and NIP rice. Values are means of three replications. Error bars indicate ± SD. Asterisks indicate significant differences between samples according to the Student’s *t*-test (*p* < 0.05). **(G)** DAB staining shows H_2_O_2_ accumulation of transgenic and wild-type rice upon normal conditions. All of the experiments were repeated three times with similar results.

Supplementary Figure 3 APX activity was affected by *M. oryzae* infected. The APX enzyme activity in indicated lines upon Guy11 treatment. Values are means of three replications. The error bars indicate ± SD. The asterisks indicate significant differences between samples according to the Student’s *t*-test (*p* < 0.05). The experiment was repeated three times with similar results.

Supplementary Figure 4 *OsAPX1* do not affect JA signaling pathway. The expression levels of JA signaling pathway relative genes are compared between OsAPX1 transgenic rice and wild type rice by qRT-PCR. Values are means of three replications. Error bars indicate + SD. ns indicates no significant difference between samples according to the Student’s *t*-test (*p* < 0.05). The experiment was repeated three times with similar results.
