# Supplementary figures and images for: OsAPX1 Positively Contributes to Rice Blast Resistance
Source: Front Plant Sci. 2022 Mar 21;13:843271. doi: 10.3389/fpls.2022.843271 (PMC8978999; doi:10.3389/fpls.2022.843271)

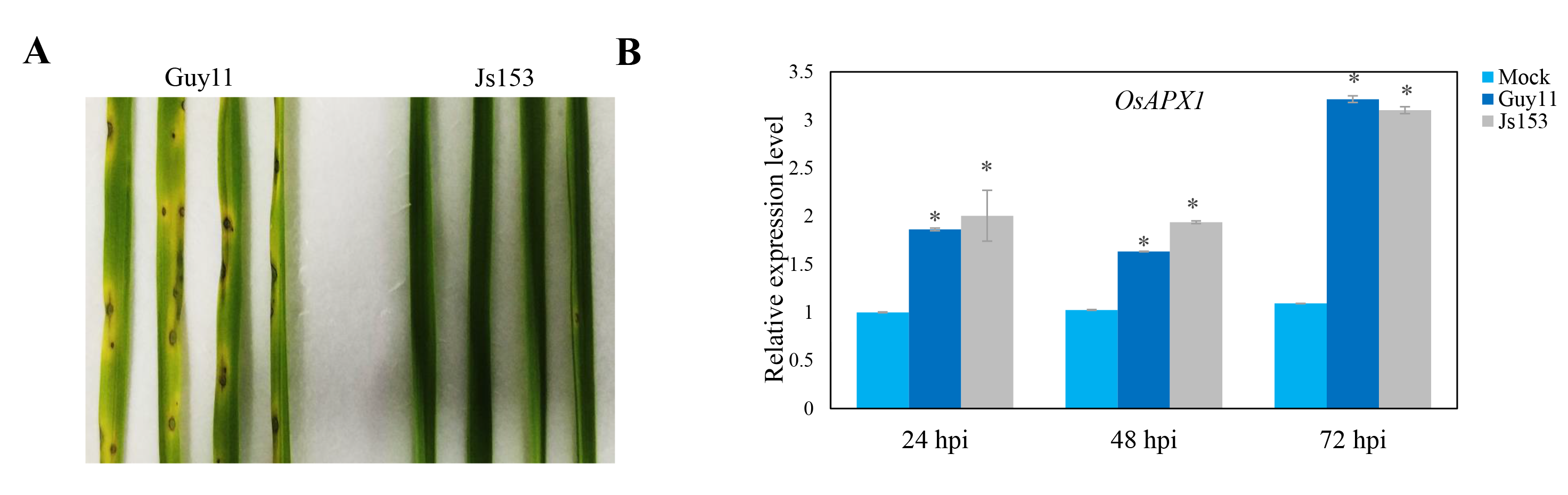

Supplement: Supplementary Figure 1 — OsAPX1 transcript induces by M. oryzae infection. (A) Rice (Oryzae sativa cv. Nipponbare) infected with strain Guy11 and Js153. The disease symptom is recorded at 72 hpi. (B) Relative transcriptional level of OsAPX1 in rice infected by mock, Guy11, and Js153 at 24, 48, and 72 hpi (OsAPX1/18srRNA). Values are means of three replications. Error bars indicate ± SD. Asterisks indicate significant differences between samples according to the Student’s t-test (p < 0.05). All of the experiments were repeated three times with similar results. [file Image_1.TIF]

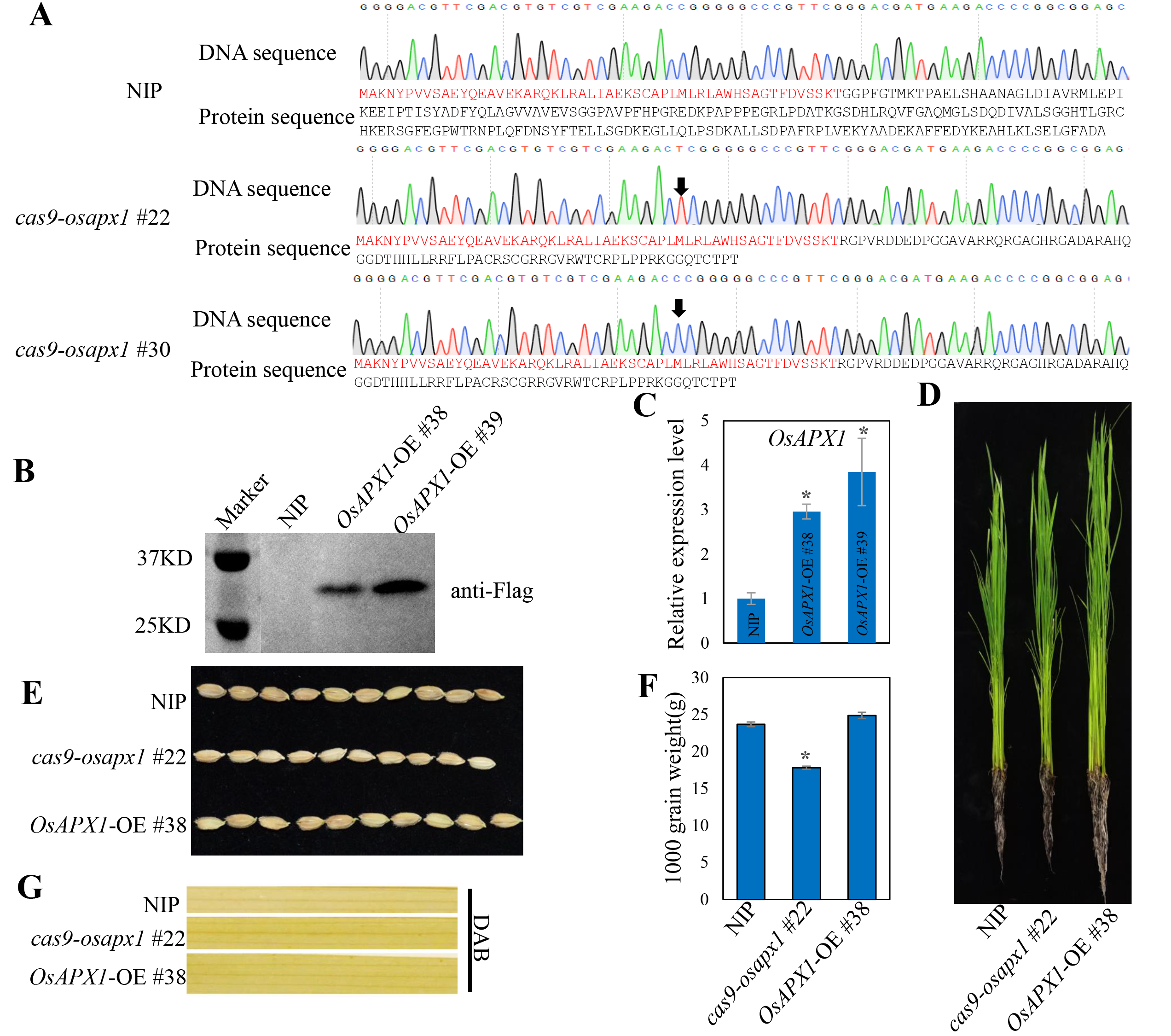

Supplement: Supplementary Figure 2 — Validation of OsAPX1 transgenic rice. (A) Sequence confirmation of the homogenous cas9-osapx1 mutant lines. (B) Protein confirmation of OsAPX1-OE lines. (C) OsAPX1 transcript level in OsAPX1-OE and NIP rice. Values are means of three replications. Error bars indicate ± SD. Asterisks indicate significant differences between samples according to the Student’s t-test (p < 0.05). (D) The phenotype of the NIP, cas9-osapx1, and OsAPX1-OE rice at 45 days post-sowing. (E) Comparison of grain length in OsAPX1-OE, cas9-osapx1, and NIP rice. (F) Weight of 1,000 seeds of OsAPX1-OE, cas9-osapx1, and NIP rice. Values are means of three replications. Error bars indicate ± SD. Asterisks indicate significant differences between samples according to the Student’s t-test (p < 0.05). (G) DAB staining shows H2O2 accumulation of transgenic and wild-type rice upon normal conditions. All of the experiments were repeated three times with similar results. [file Image_2.TIF]

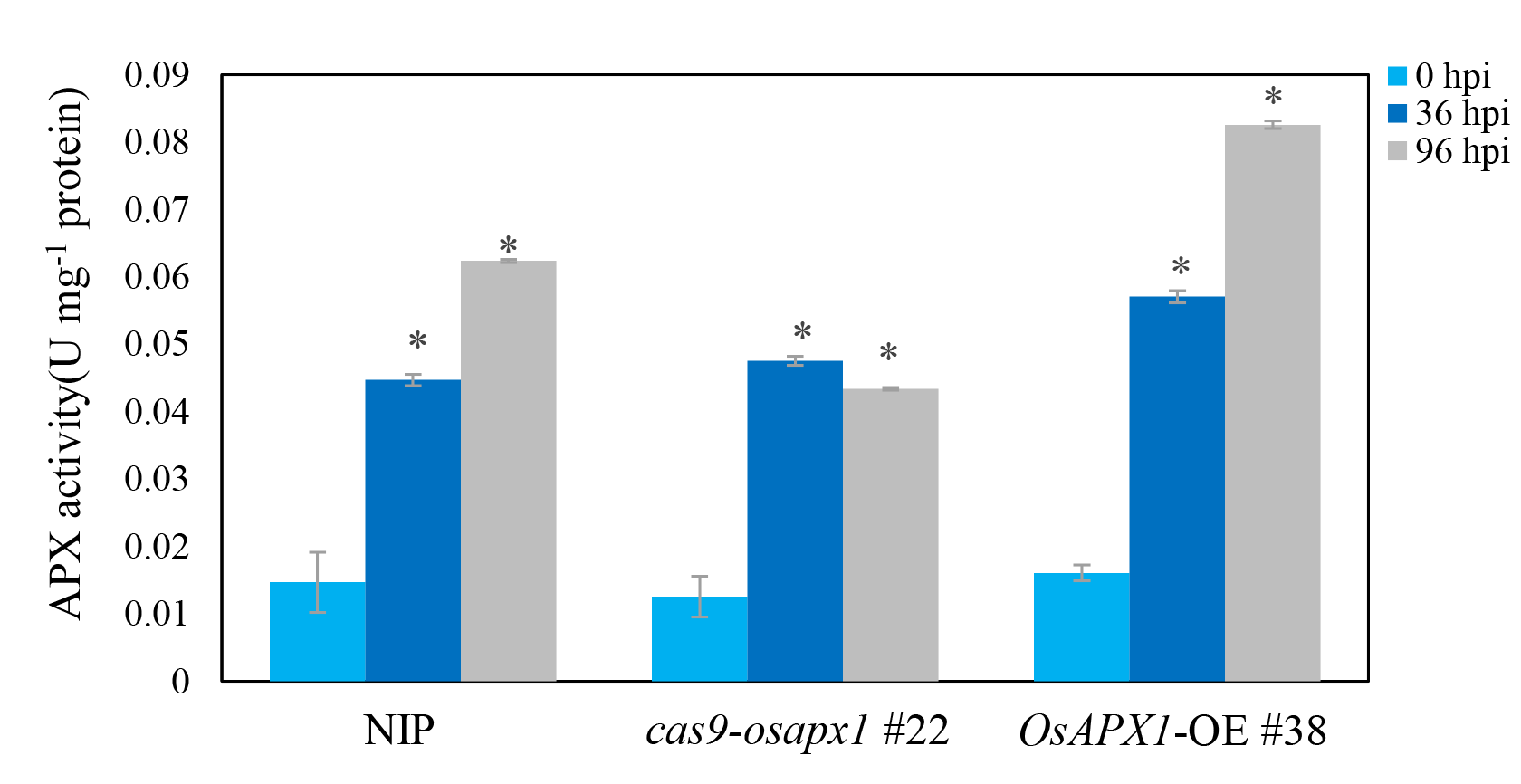

Supplement: Supplementary Figure 3 — APX activity was affected by M. oryzae infected. The APX enzyme activity in indicated lines upon Guy11 treatment. Values are means of three replications. The error bars indicate ± SD. The asterisks indicate significant differences between samples according to the Student’s t-test (p < 0.05). The experiment was repeated three times with similar results. [file Image_3.TIF]

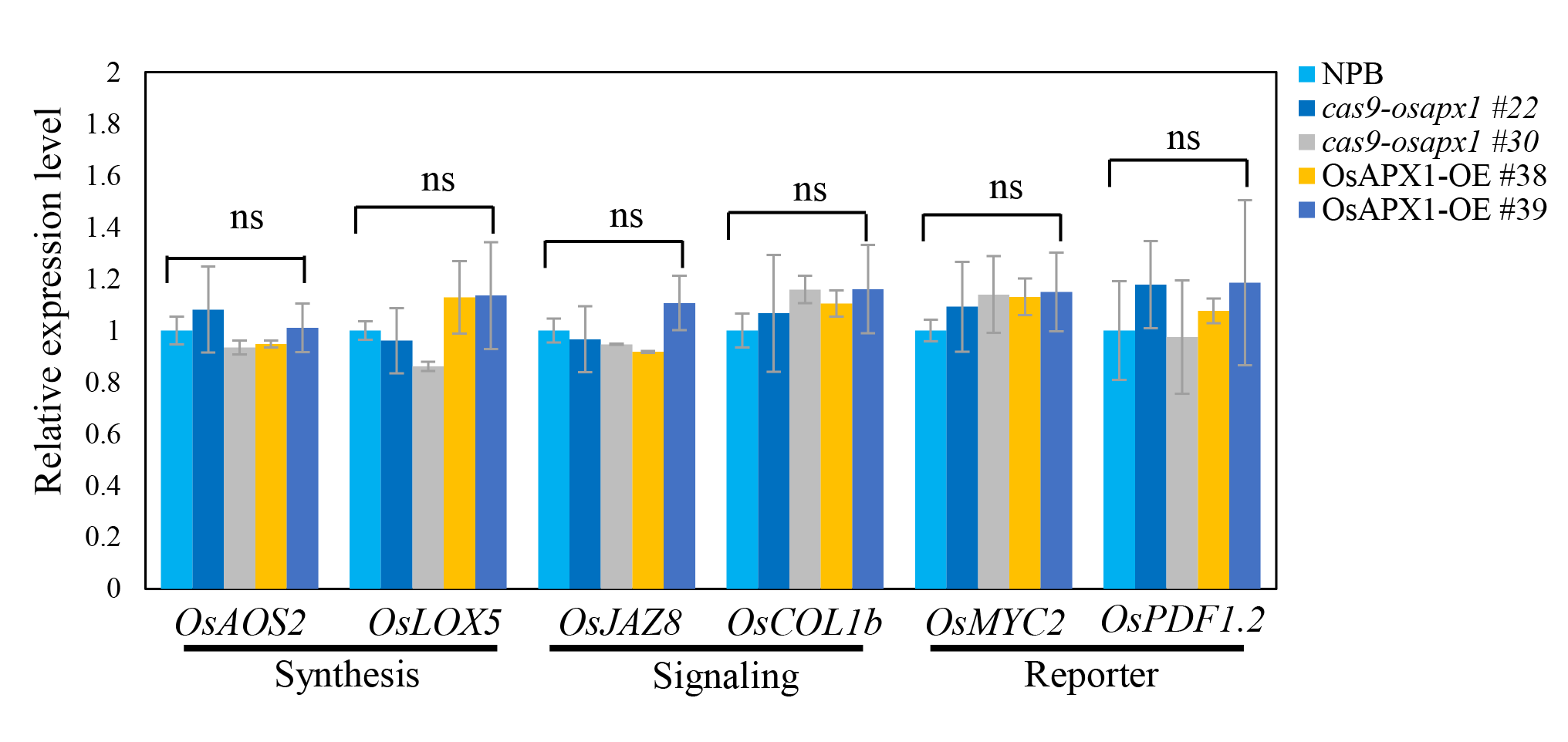

Supplement: Supplementary Figure 4 — OsAPX1 do not affect JA signaling pathway. The expression levels of JA signaling pathway relative genes are compared between OsAPX1 transgenic rice and wild type rice by qRT-PCR. Values are means of three replications. Error bars indicate + SD. ns indicates no significant difference between samples according to the Student’s t-test (p < 0.05). The experiment was repeated three times with similar results. [file Image_4.TIF]
