# Supplementary material for: OsAPX1 Positively Contributes to Rice Blast Resistance
Source: Front Plant Sci. 2022 Mar 21;13:843271. doi: 10.3389/fpls.2022.843271 (PMC8978999; doi:10.3389/fpls.2022.843271)
Supplement: Supplementary file 5 [file Table_1.DOCX]

**Table S1. The primers used in this research**

| **Primer ID** | **Primer sequence**(5’-3’) | **Objective** |
| --- | --- | --- |
| OsLOX5-QF | CTCCACCTCCACCAACATC | qRT-PCR |
| OsLOX5-QR | CTCTGAACCACGAGAACCTATC | qRT-PCR |
| OsCOL1b-QF | CCTTCAAGACTGTGCTCCTTAG | qRT-PCR |
| OsCOL1b-QR | GTACTGCAAGTCCAGCTTCTT | qRT-PCR |
| OsJAZ8-QF | GTTACCCACCTCAGCCTCAC | qRT-PCR |
| OsJAZ8-QR | TTTATACGGCGAAACCGAAC | qRT-PCR |
| OsPDF1.2-QF | AAGCCGAGCAGCCATTT | qRT-PCR |
| OsPDF1.2-QR | ATGCAGCGTCGAGTCAAG | qRT-PCR |
| OsNPR1-QF | CGTCTCCTTGATGTCCTTGATAA | qRT-PCR |
| OsNPR1-QR | GGTTTGACCGGACTACCATATC | qRT-PCR |
| OsPR1a-QF | GTGAGCTGGGACGACAC | qRT-PCR |
| OsPR1a-QR | GAAGATGTTCTCGCCGTACTT | qRT-PCR |
| OsPR1b-QF | CAGGTAGAATCATCGACCGTAAG | qRT-PCR |
| OsPR1b-QR | CGGCTACCATGGCCAAA | qRT-PCR |
| OsICS1-QF | AGTGGCTGCACGCGCAG | qRT-PCR |
| OsICS1-QR | CGAGCTCTCTTCAAGCTCATT | qRT-PCR |
| OsMYC2-QF | CCCATGGACATGAAGGACTC | qRT-PCR |
| OsMYC2-QR | TTCTCGAAGTGCTGGATCTG | qRT-PCR |
| OsAPX1-QF | CATCTCCTACGCCGATTTCTAC | qRT-PCR |
| OsAPX1-QR | CCTTGGTAGCATCAGGAAGAC | qRT-PCR |
| OsPAD4-QF | CGGTGCTTTGTGTCACATTC | qRT-PCR |
| OsPAD4-QR | GACATTGAGTGGGCAGAAGA | qRT-PCR |
| OsPAL1-QF | GTGGACATCCTCAAGCTCAT | qRT-PCR |
| OsPAL1-QR | CTTGACGGAGCTCTTGATGT | qRT-PCR |
| OsWRKY45-F | AGGAGATCCAAAACTCCAAG | qRT-PCR |
| OsWRKY45-R | TCAAAAGCTCAAACCCATAAT | qRT-PCR |
| OsAOS2-QF | CAATACGTGTACTGGTCGAATGG | qRT-PCR |
| OsAOS2-QR | AAGGTGTCGTACCGGAGGAA | qRT-PCR |
| OsPBZ1-QF | CTACTATGGCATGCTCAAGAT | qRT-PCR |
| OsPBZ1-QR | ATAGAAAGGCACATAAACACAA | qRT-PCR |
| OsRBOHa-QF | ATCCGCAAAATAAGCACCTCT | qRT-PCR |
| OsRBOHa-QR | CAGTAGCCCATCACATCAAAGA | qRT-PCR |
| OsRBOHb-QF | GGCTTCAATGCCTTCTGGT | qRT-PCR |
| OsRBOHb-QR | ATGGCTCCTAAACAACCGA | qRT-PCR |
| OsRBOHc-QF | CCAGTGGGTGGGAAAAGTG | qRT-PCR |
| OsRBOHc-QR | GTCCGATTGGCGGGTAAA | qRT-PCR |
| OsRBOHd-QF | CACAAGGTTATCGCACTGAC | qRT-PCR |
| OsRBOHd-QR | AGCGATGAGTATGTTGGTTG | qRT-PCR |
| OsRBOHe-QF | TCAAGGCAGCGATTTACCC | qRT-PCR |
| OsRBOHe-QR | CTCGCAAGCCTTCCCAAA | qRT-PCR |
| OsRBOHf-QF | CTTTCTCCATCACTTCAGCA | qRT-PCR |
| OsRBOHf-QR | GGGCCATCTACAAGCAACC | qRT-PCR |
| OsRBOHg-QF | GTCAAATGCTTATGCTGTCA | qRT-PCR |
| OsRBOHg-QR | TGTCCAGTCTCCGTTTGTT | qRT-PCR |
| OsRBOHh-QF | TACTTCGGGCAGACACGGAT | qRT-PCR |
| OsRBOHh-QR | GCGGGTTGCTGTCACTAAG | qRT-PCR |
| OsRBOHi-QF | ACCTTACCTGCGATTTTCCA | qRT-PCR |
| OsRBOHi-QR | ACCTTACCTGCGATTTTCCA | qRT-PCR |
| OsAO-QF | CACCCTCACGCTCACATAAA | qRT-PCR |
| OsAO-QR | CCTAACATTGGGAAGCAGGAT | qRT-PCR |
| OsGLO1-QF | GATCTACGACTACTACGCCTCT | qRT-PCR |
| OsGLO1-QR | GCCACTTCACATCCTTCCA | qRT-PCR |
| Os18s rRNA-QF | ATGATAACTCGACGGATCGC | qRT-PCR |
| Os18s rRNA-QR | CTTGGATGTGGTAGCCGTTT | qRT-PCR |
| MoPot2-QF | ACGACCCGTCTTTACTTATTTGG | qRT-PCR |
| MoPot2-QR | AAGTAGCGTTGGTTTTGTTGGAT | qRT-PCR |
| OsUbq-QF | TTCTGGTCCTTCCACTTTCAG | qRT-PCR |
| OsUbq-QR | ACGATTGATTTAACCAGTCCATGA | qRT-PCR |
| cas9-apx1-u6aF | GCCGTTCGGGACGATGAAGACCC | Transgenic Construction |
| cas9-apx1-u6aR | AAACGGGTCTTCATCGTCCCGAA | Transgenic Construction |
| cas9-apx1-u6bF | GTTGCTGTCGCACGCCGCCAACG | Transgenic Construction |
| cas9-apx1-u6bR | AAACCGTTGGCGGCGTGCGACAG | Transgenic Construction |
| OsAPX1-OE-F | CTCGGTACCCGGGGATCCATGGCTAAGAACTACCCCGTCG | Transgenic Construction |
| OsAPX1-OE-R | CAGGTCGACTCTAGAGGATCCTTAAGCATCAGCGAACCCCAG | Transgenic Construction |
|  |  |  |
|  |  |  |
